# Integrated Method to Attach DNA Handles and Functionally Select Proteins to Study Folding and Protein-Ligand Interactions with Optical Tweezers

**DOI:** 10.1038/s41598-017-11214-z

**Published:** 2017-09-07

**Authors:** Yuxin Hao, Clare Canavan, Susan S. Taylor, Rodrigo A. Maillard

**Affiliations:** 1 Department of Chemistry, Georgetown University, Washington, DC 20057 USA; 2 Department of Pharmacology & Department of Chemistry and Biochemistry, University of California, San Diego, La Jolla, CA 92093 USA

## Abstract

Optical tweezers has emerged as a powerful tool to study folding, ligand binding, and motor enzymes. The manipulation of proteins with optical tweezers requires attaching molecular handles to the protein of interest. Here, we describe a novel method that integrates the covalent attachment of DNA handles to target proteins with a selection step for functional and properly folded molecules. In addition, this method enables obtaining protein molecules in different liganded states and can be used with handles of different lengths. We apply this method to study the cAMP binding domain A (CBD-A) of Protein kinase A. We find that the functional selection step drastically improves the reproducibility and homogeneity of the single molecule data. In contrast, without a functional selection step, proteins often display misfolded conformations. cAMP binding stabilizes the CBD-A against a denaturing force, and increases the folded state lifetime. Data obtained with handles of 370 and 70 base pairs are indistinguishable, but at low forces short handles provide a higher spatial resolution. Altogether, this method is flexible, selects for properly folded molecules in different liganded states, and can be readily applicable to study protein folding or protein-ligand interactions with force spectroscopy that require molecular handles.

## Introduction

Single molecule optical tweezers is a powerful force spectroscopy technique to investigate molecular mechanisms of protein and nucleic acid folding^1–9^, ligand binding^10^, enzyme catalysis^11, 12^ and motor proteins^13–19^. In optical tweezers experiments, force is applied to a target protein that is tethered between two beads or between a bead and a glass slide^20, 21^. Tethering of the protein is enabled by the covalent attachment of molecular handles. Molecular handles not only serve as force transducers, but they also prevent unspecific and undesired interactions between the target protein and the surface of the bead or slide. The most commonly used molecular handles are double-stranded DNA (dsDNA)^9, 22, 23^. In recent years, various experimental strategies to covalently link dsDNA handles to target proteins have been developed. A pioneering and widely-used strategy to attach dsDNA handles is via disulfide bond linkage to cysteine residues in the target protein^1, 2, 10, 24^. More recent approaches involve enzymatic reactions between a fusion protein or a peptide tag, which are engineered to the target protein, and the according enzymatic substrate that is covalently linked to the dsDNA handle^3, 8, 10, 14, 15, 25–28^.

Among the many strategies to covalently attach dsDNA handles to a target protein, disulfide bonds have important advantages for protein folding studies. First, they provide flexibility on the position of the handle attachment, allowing the examination of the protein’s energy landscape along different reaction coordinates^29, 30^. Second, with disulfide bond linkages it is possible to selectively manipulate a specific region or domain within the target protein, enabling the investigation of high-order protein function such as folding cooperativity between coupled domains^2^. However, a concern in protein folding studies with optical tweezers is the difficulty of selecting those protein molecules that are functional and therefore properly folded. This is because during an experiment, proteins are selected by the interactions between the tags in the dsDNA handles and the bead or slide. Thus, a protein sample preparation that contains an aggregated or misfolded population may lead to artifacts and wrong interpretations of the native behavior of the protein.

**Figure 1:**
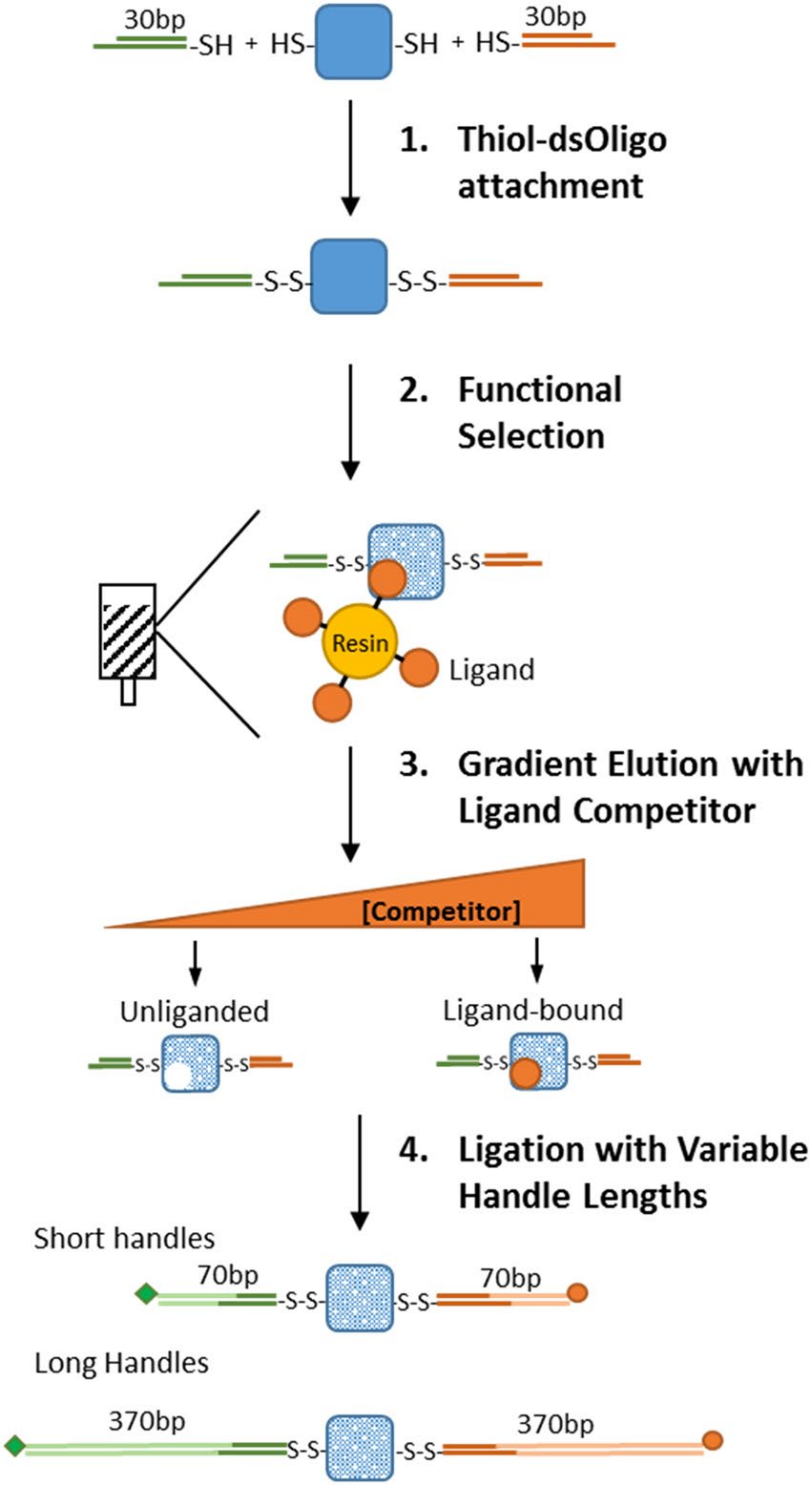
Combined method to attach dsDNA handles and select for functional protein molecules. After its purification, the target protein is covalently linked with thiol-modified dsOligos (step 1). The protein-oligo chimera is functionally selected using a ligand-coupled resin (step 2). Functional and properly folded proteins are eluted using a concentration gradient of a ligand competitor, generating different liganded protein states (step 3). Ligation of biotin- and digoxigenin-modified dsDNA handles of variable lengths, as required by the experimental set-up (step 4).

In this study, we developed a methodology that integrates the covalent attachment of dsDNA handles to proteins via disulfide bonds with a selection step of functional and properly folded proteins (Fig. 1). Briefly, we first crosslink short thiol-modified oligos (~30 bp) to the target protein to generate a protein-oligo chimera. Because short oligos can be easily prepared in high concentrations (~2 mM), the yield of the protein-oligo chimera is very high. Then, we select functional molecules from the protein-oligo mixture by using an agarose resin coupled to the corresponding protein ligand. Elution of functional molecules is achieved by using a gradient concentration of a competitor ligand. A gradient elution enables obtaining protein-oligo chimeras in different states, i.e. unliganded and bound states. Lastly, we ligate dsDNA handles functionalized with either biotin or digoxigenin to the functionally selected protein-oligo chimera. The dsDNA handles can be tailored to a specific length, thereby providing flexibility for different optical tweezers experimental set-ups.

We apply this method to the cAMP binding domain A (CBD-A) of the regulatory subunit of protein kinase A (PKA). We covalently attached dsDNA handles of either 370 bp or 70 bp to the protein-oligo chimera, and show that the mechanical fingerprints of the CBD-A are indistinguishable between the two handle lengths. However, at low forces the data obtained with 70-bp dsDNA handles has a significantly higher spatial resolution than the data obtained with 370-bp handles. Importantly, the functional selection step with a homemade cAMP-coupled agarose revealed more reproducible and consistent unfolding data. In contrast, without the functional selection step, the protein often displays misfolded events. We used a gradient of cAMP concentration during the elution of functional proteins to study the CBD-A in unliganded and cAMP-bound states. Altogether, this method is flexible in handle length, high-yield, selects for functional protein molecules, and is readily applicable to study protein folding or protein-ligand interactions with force spectroscopy techniques requiring molecular handles.

## Results

### High Yield Production of a Protein-Oligo Chimera

**Figure 2:**
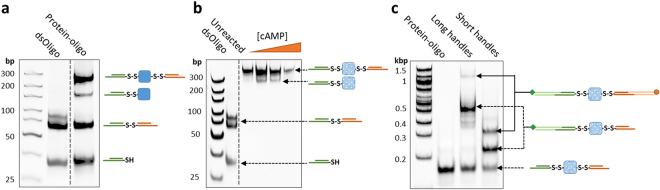
Monitoring the dsDNA handle attachment reaction and functional selection of the CBD-A. (**a**) 5′**-**Thiol-modified dsOligos were attached to the CBD-A. (**b**) The protein-oligo chimera was functionally selected and eluted from a cAMP-coupled agarose using a step-wise gradient concentration of cAMP: 0.02 mM, 0.2 mM, 2 mM and 20 mM. (**c**) The selected protein-oligo chimera was ligated with long (370 bp) or short (30 bp) dsDNA handles modified with digoxigenin (green square) and biotin (orange circle). All gels are native acrylamide gels stained with ethidium bromide. Dashed lines correspond to gel areas or lanes that were empty and thus cropped from the original gels for visual simplicity (see Supplementary Figure 1 for complete gels and experimental details).

Two cysteine residues were engineered into the CBD-A at positions 110 and 243 (numbering based on the full-length regulatory subunit RIα of Protein Kinase A)^31^. Thiol-modified double-stranded oligos (referred as dsOligos) were attached to the cysteine-modified protein via disulfide bond linkage. The intense upper band demonstrates that the majority of the protein is covalently modified with two dsOligos (Fig. 2a). The high yield of formation of the protein-oligo chimera can be attributed to the high concentration of all the reactants, which were about 200 μM. Given that the CBD-A reacted with two dsOligos with unique restriction sites (RS1 and RS2) in equimolar concentrations, it is expected that 50% of molecules in the protein-oligo chimera sample is modified with the required configuration for optical tweezers, namely, one dsOligo with RS1 corresponding to the biotin-modified handle, and one dsOligo with RS2 corresponding to the digoxigenin-modified handle. Other species present in the sample may have either two RS1 or two RS2, but are unable to form a tether between the two beads in the optical tweezers set-up.

### Selection of Functional Proteins for Optical Tweezers Experiments

After crosslinking the dsOligos to the CBD-A, we performed a functional selection step using a homemade cAMP-coupled agarose. To study protein-ligand interactions with optical tweezers, we obtained samples in the unliganded and cAMP-bound conformations. This is achieved by eluting the protein-oligo chimera from the cAMP-coupled agarose using a broad range of cAMP concentrations that covered three orders of magnitude, from 0.02 mM to 20 mM (Fig. 2b). Since the final sample is diluted 10^6^–10^7^ times before applying it to the optical tweezers, the cAMP concentration of the sample eluted with [cAMP] = 0.02 mM is in the picomolar range, which is below the dissociation constant (Kd ~ 3 nM)^32, 33^. Likewise, the sample eluted with [cAMP] = 20 mM has a final cAMP concentration in the nanomolar range, and can be used to study the protein in the cAMP-bound state.

After the functional selection step, the protein-oligo chimera was ligated with modified dsDNA handles that contain complementary overhangs. Because the ligation step occurs after the covalent modification of the target protein, the dsDNA handles can be tailored to a specific length. Shown in Fig. 2c are the ligations using two handle lengths: 370 bp and 70 bp dsDNA handles.

### Single Molecule Data with Functionally Selected Proteins is More Homogeneous and Reproducible

Previous studies have used electro-elution or affinity purification using a histag to remove competing species that can form a tether in the optical tweezers, i.e., species made with two thiol-modified handles but with no protein^22, 23^. The functional selection step used in this study not only removed competing species but also selected for protein molecules that were ligand-binding competent after attaching dsDNA handles. For the CBD-A, we found that as much as 10–40% of the sample is unable to bind the cAMP-resin after attaching the thiol-modified oligos. This percentage, however, varies between CBD constructs or mutants (data not shown).

**Figure 3:**
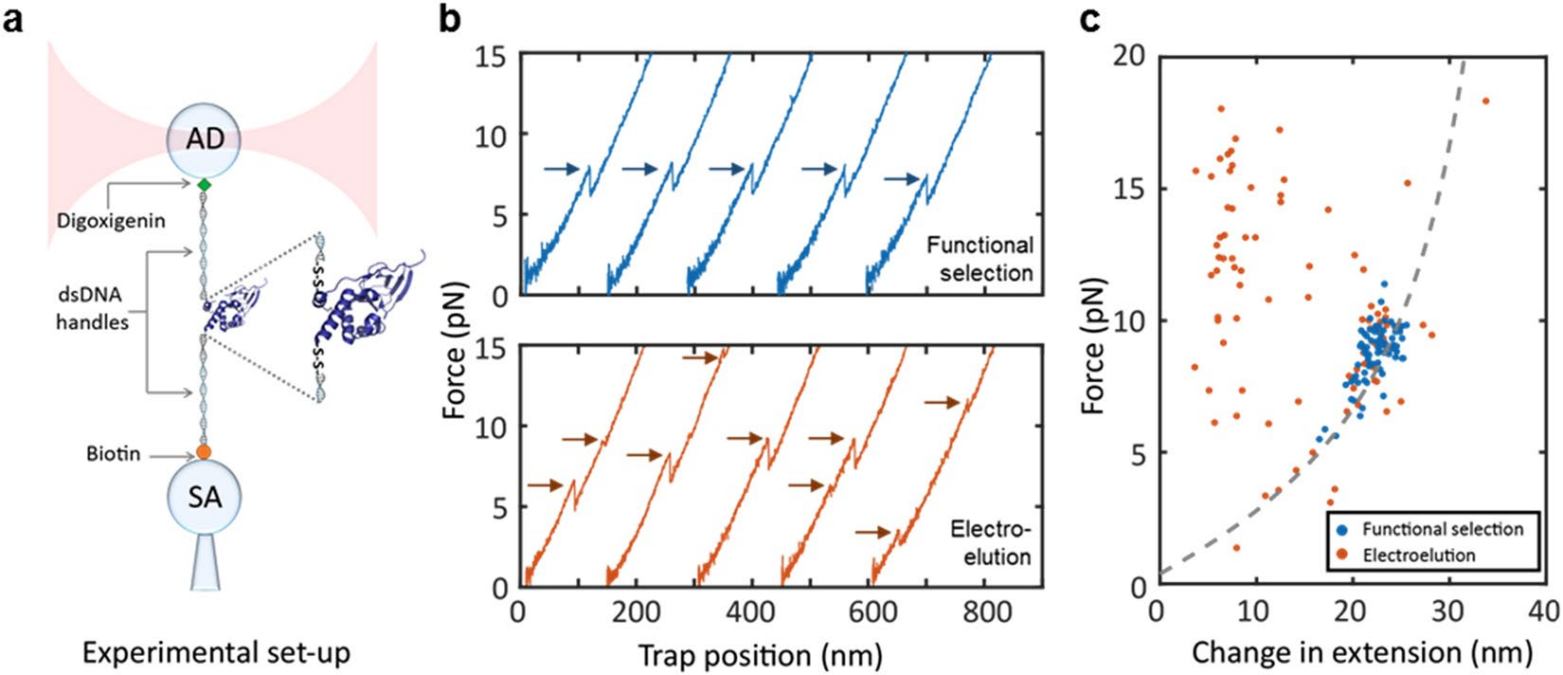
Single molecule experiments with the CBD-A. (**a**) Schematic representation of the optical tweezers experimental set-up. (**b**) Representative force-extension curves of the CBD-A in the unliganded state. Protein samples were purified by functional selection (top panel) and by electro-elution (bottom panel). (**c**) Change in extension upon unfolding vs. force for functionally selected (blue) and electro-eluted (orange) samples. The gray dashed line shows the theoretical Worm Like Chain (WLC) model of the unfolded CBD-A, using a contour length of 46.5 nm and a folded distance of 2 nm.

To investigate the effect of sample preparation at the single molecule level, we generated force-extension curves of the CBD-A in the unliganded state using samples that were either functionally selected or samples that were only purified by electro-elution (Fig. 3a). The functionally selected sample was eluted from the cAMP-coupled resin using [cAMP] = 0.02 mM and was subsequently diluted ~10^7^ times before applying it to the optical tweezer. This sample therefore had an approximate [cAMP] = 2 pM, which is three orders of magnitude lower than the reported dissociation constant^32, 33^. Force-extension curves using the functionally selected sample always displayed a single unfolding transition in a narrow force range between 5–10 pN (Fig. 3b, top). Moreover, the change in contour length upon unfolding (ΔLc) was 45 ± 3 nm (mean ± standard deviation), in agreement with the expected contour length increase based on the Worm Like Chain model (WLC)^34^ (CBD-A: 133 amino acids × 0.365 nm − 2 nm (folded distance) = 46.5 nm) (Fig. 3c). In contrast, samples that were only electro-eluted showed single or double unfolding transitions, and in a wide force range between 4–25 pN (Fig. 3b, bottom). Furthermore, only 40% of electro-eluted protein molecules showed single unfolding events that matched the expected WLC, indicating that the other 60% of proteins were not properly folded. These misfolded or damaged proteins can originate either from the DNA-handle attachment process or the electro-elution step. However, the functional selection step can eliminate this issue by selecting properly folded proteins.

### Mechanical Fingerprints of the CBD-A in the Absence and Presence of cAMP

**Figure 4:**
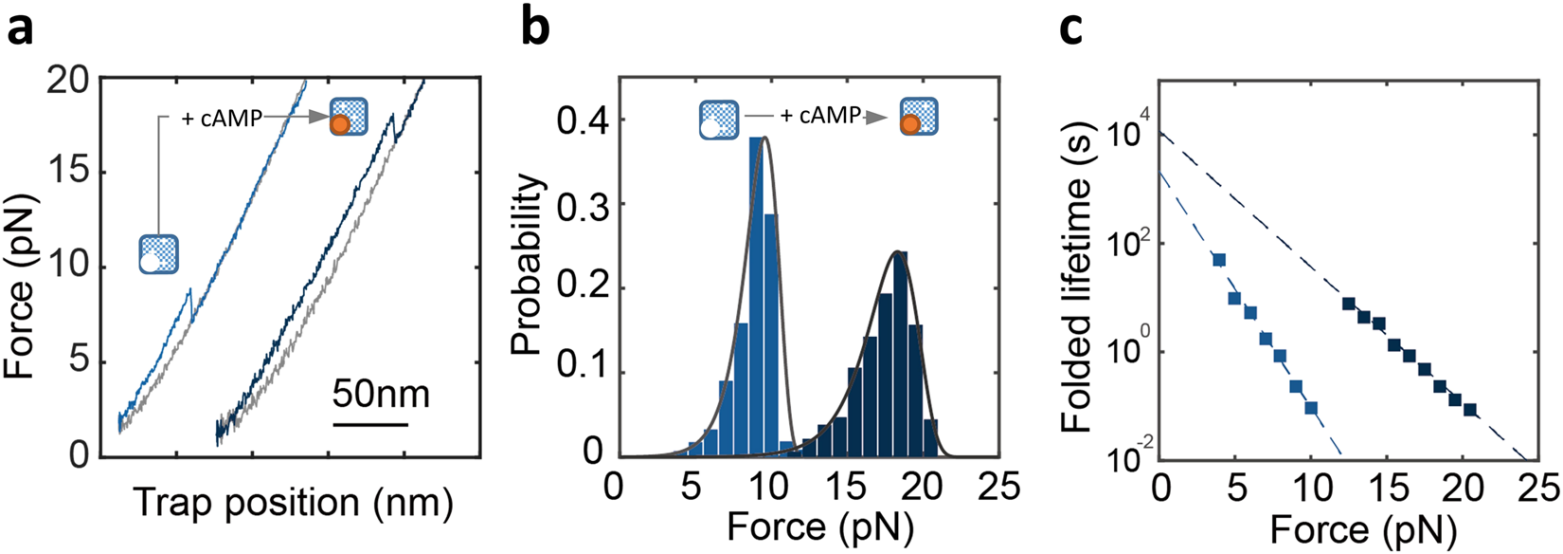
Kinetic analysis of CBD-A and cAMP interactions. (**a**) Representative force-extension curve of the CBD-A in the absence (left) and in the presence of [cAMP] = 0.5 mM (right). (**b**) Unfolding force probability distribution for the CBD-A in the absence (light blue, N = 1157) and in the presence of cAMP (dark blue, N = 829). The solid line represents the unfolding force distribution reconstructed from the force-dependent lifetimes (see main text). (**c**) Folded state lifetimes (*τ*_folded_) as a function of force extracted from the unfolding force distributions in the absence (light blue squares) and presence of cAMP (dark blue squares). The dashed line corresponds to the fit of Bell’s model to the force-dependent rates.

Force-extension curves obtained in the absence and presence of cAMP (0.5 mM) showed important differences (Fig. 4a). The unfolding force distribution for the unliganded CBD-A had a maximum at ~9 pN, whereas for the cAMP-bound state the unfolding force distribution peaked at ~ 18 pN (Fig. 4b). Clearly, the additional interactions established between the cyclic nucleotide binding pocket and cAMP^35^ have a profound effect on the mechanical stabilization of the CBD-A.

To further dissect the effect of cAMP binding to the CBD-A, we used the method developed by Dudko *et al.* ^36^ to transform the unfolding force distributions into folded state lifetimes as a function of force (Fig. 4c). This analysis enabled us to determine the unfolding rate (inverse of the folded state lifetime) extrapolated to zero force (k_unf_) and the distance to the transition state (Δx^‡^). For the unliganded state, we obtained k_unf_ = 4.6·10^−4^ s^−1^ and Δx^‡^ = 4.1 nm. Interestingly, the observed Δx^‡^ is similar to those reported for proteins in the molten globule state^7^, suggesting that the CBD-A conformation in the unliganded state is deformable against mechanical force. For the cAMP-bound state we observed that k_unf_ is 5 times lower (8.3·10^−5^ s^−1^) while Δx^‡^ is almost two times shorter (2.4 nm). This indicates that cAMP binding not only decreases the rate of unfolding of the CBD-A but also makes the protein more brittle and likely more compact.

Additional experiments will be needed to fully describe how the energy landscape of the CBD-A changes upon cAMP binding. Importantly, these two data sets were obtained from the same sample preparation that was eluted from the cAMP-coupled agarose with different cAMP concentrations.

### Flexibility of DNA Handle Length for Optical Tweezers Experiments

**Figure 5:**
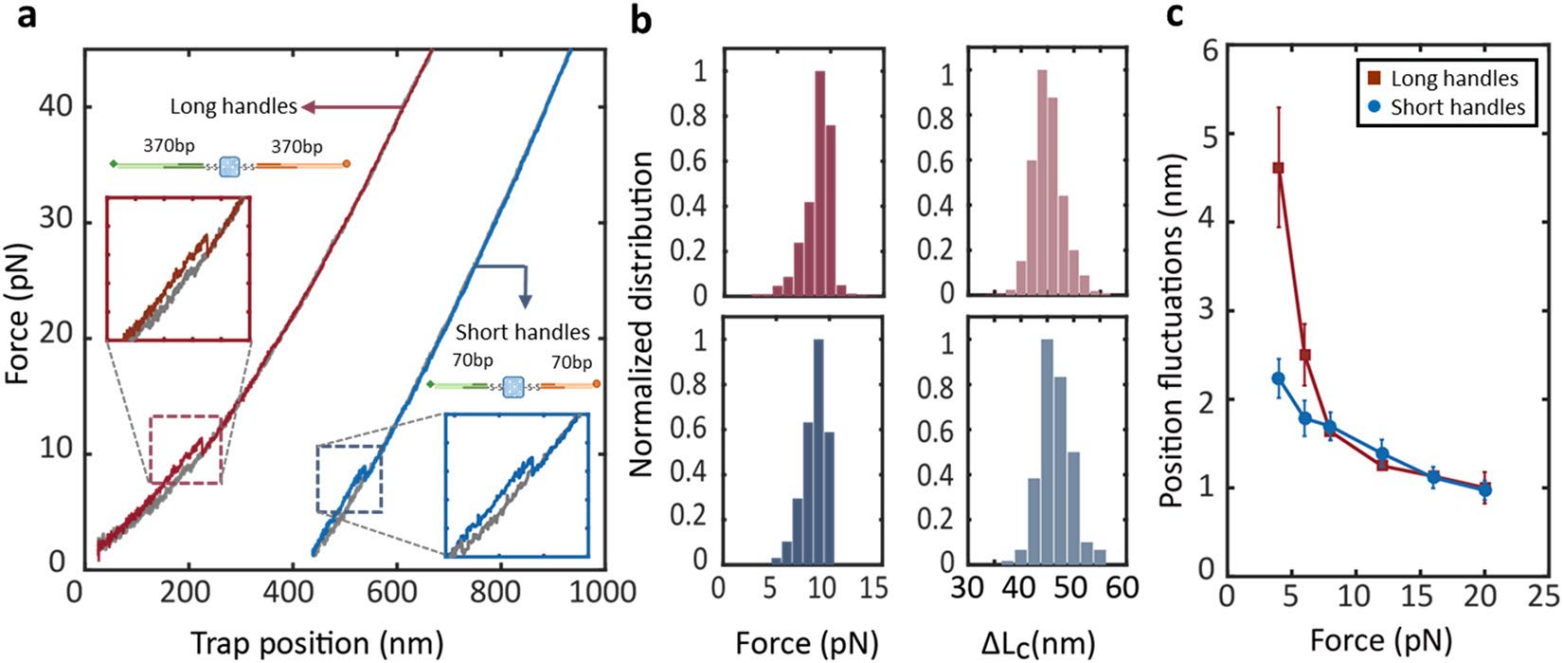
Comparison of mechanical fingerprints and spatial resolution using variable dsDNA handle lengths. (**a**) Representative force-extension curves of the CBD-A with long (370 bp, red) and short (30 bp, blue) dsDNA handles. (**b**) Normalized distributions corresponding to unfolding forces and changes in contour length upon unfolding for the CBD-A using long (top panel) and short (bottom panel) dsDNA handles. (**c**) The position fluctuations of long and short dsDNA handles as a function of force.

We characterized the mechanical properties of the CBD-A using long (370 bp) and short (70 bp) dsDNA handles. The unfolding trajectories with both long and short dsDNA handles are shown in Fig. 5a. Both trajectories show a small rip at ~9 pN that corresponds to the mechanical unfolding of a single CBD-A (Fig. 5a, inset). The unfolding force distributions (Fig. 5b, left) as well as the mean contour length (Fig. 5b, right) increase upon unfolding were indistinguishable between long and short handles.

At low forces, we observed that short dsDNA handles have a larger stiffness (0.070 pN/nm) compared to long dsDNA handles (0.063 pN/nm). The larger stiffness likely contributes to a higher signal-to-noise ratio for short dsDNA handles, especially at forces below 7 pN (Fig. 5c). In fact, at forces between 3–5 pN, the position fluctuations of long dsDNA handles are twice as much as the fluctuations for short dsDNA handles. As the force between the tether increases, the fluctuation difference between long and short dsDNA handles is narrowed, and completely disappears at forces >8 pN.

## Discussion

In this study, we integrated the covalent attachment of dsDNA handles with a selection step of functional proteins. This combined approach provides several advantages to study protein folding and protein-ligand interactions using single molecule mechanical manipulation techniques. First, it removes unreacted and unwanted byproducts, which makes data collection much more efficient. Second, it selects functional protein molecules, i.e., properly folded proteins bind to the cAMP-coupled agarose. Third, by eluting the sample using a gradient of cAMP concentrations, it is also possible to study the unliganded and cAMP-bound states of the protein using the same sample preparation. Noteworthy, we show that this approach provides an avenue to directly measure tight protein-ligand interaction with dissociation constants in the nanomolar to picomolar range, which are typically difficult to monitor using bulk biophysical methods. Here we apply this method to investigate protein-ligand interactions using the cAMP binding domain A of the regulatory subunit of protein kinase A as the model system.

In previous studies, proteins covalently linked with dsDNA handles have been purified by electro-elution or using affinity tags in the protein (i.e., histag)^23^. This purification step is an important improvement to enrich the desired protein-DNA construct. However, a purification step without selecting active proteins can lead to tethering misfolded molecules in the optical tweezers. As shown in Fig. 3b and c, the functionally selected protein sample has a consistent and reproducible behavior in both unfolding forces and the accompanying change in extension. Given the diverse choices of ligand analogs, non-hydrolysable substrates, and crosslinking reagents, this functional selection step can be applied to many other protein systems. In the case of proteins that do not interact with ligands, the use of specific monoclonal or polyclonal antibodies raised against a conformational epitope could be used. Since antibodies have a high affinity for epitopes, they can be used as affinity reagents in the protein-oligo purification step. Alternatively, a binding protein partner could be used to achieve functional selection via protein-protein interactions, provided that the affinity between them is high.

Lastly, this method also provides the flexibility of the dsDNA handle length, i.e., it is straight-forward to customize the handle length using the same protein-oligo preparation. The characterization of unfolding trajectories demonstrates that both long and short dsDNA handles are able to generate consistent results during unfolding experiments performed in optical tweezers. However, as reported by others^23^, our results show that shorter dsDNA handles have a higher spatial resolution.

## Materials and Methods

### Thiol-Modified dsOligo Preparation

We prepared two sets of modified double stranded oligos (dsOligos) with a thiol group in the 5′ end of the forward sequence, and a 5′ phosphate in the reverse complementary sequence (IDT, Inc.). Both dsOligos shared the same thiol-modified forward sequence: 5′-Thiol-GTTACGCCTATTCCTATCATATGAAGACA. However, the reverse complementary sequence had unique, non-palindromic 5′ overhangs (underlined) referred as RS1 (5′-Phosphate- CGGAGTGTCTTCATATGATAGGAATAGGCGTAAC) and RS2 (5′-Phosphate-CGACGTGTCTTCATATGATAGGAATAGGCGTAAC) for restriction sites 1 and 2, respectively.

Thiol- and phosphate-modified oligos were dissolved in annealing buffer (10 mM Tris, 10 mM EDTA, 50 mM NaCl, pH 7.4) and annealed (95 °C to 4 °C, 1 °C/min) in a Mastercycler Nexus GX2 (Eppendorf). The thiol groups of the dsOligos were deprotected in 0.17 M sodium phosphate (pH 8.0) with 40 mM dithiothreitol (DTT) overnight at 37 °C. The next day, deprotected dsOligos were purified using an Amersham PD-10 column. The purified dsOligos were concentrated by ethanol precipitation, dissolved in annealing buffer, and store at −20 °C with 40 mM DTT. A typical concentration of dsOligos was 2 mM.

### DTDP-Activation of Cysteine-Modified Proteins

The cysteine-modified CBD-A was engineered using the QuikChange site-directed mutagenesis kit from Agilent. The protein was purified as documented previously^37^ but with the addition of 5 mM DTT to all buffers. The activation of the thiol-modified protein with 2,2′-dithioldipyridine (DTDP) followed the protocol described previously^23^ with minor modifications. Briefly, prior to the covalently attachment of the dsOligos, the protein was reduced with 5 mM DTT overnight in DNA crosslinking buffer (50 mM Tris, 100 mM NaCl, pH 7.6). The next day, the protein was concentrated to ~500 μM. The excess DTT was removed by three consecutive Micro Bio-Spin 6 chromatography columns (Bio-rad) pre-equilibrated with DNA crosslinking buffer. Then, the protein was reacted with a 5–25 molar excess DTDP for 2 h at room temperature. The excess DTDP was removed using three additional Micro Biospin 6 column pre-equilibrated with DNA crosslinking buffer.

### Protein-Oligo Attachment

Both dsOligos were diluted to ~200 μM in DNA crosslinking buffer with 10 mM DTT, and incubated overnight at 37 °C. The excess of DTT was removed using three Micro Bio-Spin 6 chromatography columns. The resulting dsOligos concentration was measured spectrophotometrically, and then immediately reacted with the thiol-pyridine activated protein (Fig. 1, step 1). The molar ratio of protein and the two dsOligos was 1:1:1 with a final concentration of ~100 μM each. The reaction was incubated at 4 °C overnight.

### Functional Selection of Protein-Oligo Chimera

The mixture of protein and dsOligos was incubated with a cAMP-coupled agarose resin for 4 h at 4 °C (Fig. 1, step 2). Preparation of the resin is described by the Diller *et al*.^38^. Briefly, NHS-activated agarose 4 Fast Flow (GE Healthcare Life Science) was equilibrated with 0.1 M HCl. 8-AEA-cAMP (BIOLOG) was incubated with activated agarose resin at room temperature for 3 h in 0.2 M NaHCO_3_, 0.5 M NaCl (pH = 7.5). The reaction was blocked with 0.5 M ethanolamine (pH = 8.3) for 2 h. The resin was washed three times by alternating a high pH buffer (0.1 M Tris-HCl, pH = 8.3) and a low pH buffer (0.1 M CH_3_COONa, 0.5 M NaCl, pH = 4.8). The cAMP-coupled resin is stored in 0.05 M H_3_BO_3_, 0.5 M NaCl, 10 μM IBMX (pH = 8.5) with 20% ethanol.

The functional selection step allowed removing unreacted dsOligos while at the same time purifying proteins that were able to bind their natural ligand, namely, cAMP. To thoroughly remove the excess of unreacted dsOligos, the agarose resin was washed with DNA crosslinking buffer three times with at least 20 times the volume of the resin. The protein-oligo chimera was eluted using increasing amounts of cAMP dissolved in DNA crosslinking buffer: 0.02 mM, 0.2 mM, 2 mM and 20 mM (Fig. 1, step 3). For each elution step, the solution was incubated for 30 min. All elutions containing the protein-oligo chimera were aliquoted and stored with 30% glycerol at −20 °C. Before the optical tweezers experiments, a small aliquot of protein-oligo chimera is directly ligated to dsDNA handles of variable lengths, tailored to a specific experimental geometry (Fig. 1, step 4). Next, we describe the preparation of dsDNA handles of ~370 bp each (referred as long dsDNA handles), or 70 bp each (referred as short dsDNA handles).

### Preparation of Long dsDNA Handles

The biotin- and digoxigenin-modified dsDNA handles were generated by PCR reaction using the plasmid pPROEX HTa (Addgene) as template. A 332 bp biotin-modified dsDNA handle was prepared using the forward primer 5′-TATTATTTTCTCCCATGAAGACGGTCCGCGACTG together with reverse primer 5′-Biotin-CGGTATCGTCGTATCCCACTACC (IDT, Inc). This handle is ligated to the dsOligo labeled RS1 in the protein-oligo chimera. The 315 bp digoxigenin-modified dsDNA handle was generated using the forward primer 5′-GACGATACCGAAGACAGGTCGTGTTATATCC and reverse primer 5′-digoxigenin-CCGTGCAGTCGATGATAAGCTGTC (IDT, Inc). This handle is ligated to the dsOligo labeled RS2 in the protein-oligo chimera. The PCR products were purified using NucleoSpin Gel and PCR Clean-up kits, from Clontech Laboratories. Usually, 10–12 μg of dsDNA handles can be prepared at a time using 8 × 100 μL PCR reactions. The PCR product was further digested with BpiI enzyme (Thermos Scientific) at 37 °C overnight. BpiI recognizes the sequence GAAGAC (underlined in forward primers), however, it cuts 2 bp downstream, leaving a 4 bp non-palindromic sticky overhang. Therefore, even if both dsDNA handles are generated from the same plasmid or DNA template, BpiI provides the possibility of having different overhang sequences. The plasmid pPROEX-HTa has two GAAGAC sequences, located ~350 bp from each other. The two primer sets used in this study were designed to amplify different segments of the template.

### Preparation of Short dsDNA Handles

Short dsDNA handles were prepared by annealing two complementary oligonucleotides of 44 and 40 bases. The forward sequence is 4 bases longer than the reverse sequence to generate a sticky overhang for ligation with the protein-oligo chimera. The biotin-modified dsDNA short handle has the forward sequence 5′-Phosphate-TCCGGTGCGGATATCTCGGTAGTGGGATACGAC GATACCG and the reverse sequence is 5′-Biotin-CGGTATCGTCGTATCCCACTACCGAGA TATCCGCAC. This handle ligates to the dsOligo labeled RS1 in the protein-oligo chimera.

The digoxigenin-modified dsDNA short handle has the forward sequence 5′-Phosphate-GTCGTTATCTGGTTTGACAGCTTATCATCGACTGCACGG and the reverse sequence 5′-digoxigenin-CCGTGCAGTCGATGATAAGCTGTCAAACCGATCAA. This handle ligates to the dsOligo labeled RS2 in the protein-oligo chimera. Both oligos were diluted in 2X annealing buffer (20 mM Tris, pH 7.5–8.0, 100 mM NaCl, 2 mM EDTA) and annealed to a final concentration of 100 μM.

### Ligation of dsDNA Handles to Protein-Oligo Chimera

Long or short handles can be directly ligated to the protein-oligo chimera. By using non-palindromic overhang sequences there is no ligation between the handles themselves, which otherwise can make a tether in the optical tweezers and decreases the efficiency of data collection.

Usually, about 150 ng of each dsDNA handle is needed for a 20 μL ligation. We used a homemade 10X ligation buffer that consists of 400 mM Tris-HCl, 100 mM MgCl_2_ and 5 mM ATP (pH 7.8 at 25 °C). The 100 mM DTT typically seen in commercial ligation buffers was omitted to prevent the reduction of the disulfide linkage between the protein and dsOligos. Here, we find that the ideal ligation ratio is 1:1:1 for protein-oligo chimera and each handle.

### Optical Tweezers Experiments and Data Analysis of Force-Extension curves

The ligation product was diluted ~1000-fold in DNA crosslinking buffer. We then take 1 μL of the diluted ligation product and mix it with 3 μL of 3.1 μm polystyrene beads (Spherotech) coated with anti-digoxigenin antibodies for 5 min at room temperature. The sample is further diluted to 1 mL before applying it to the optical tweezers. The optical tweezer experiments were performed in DNA crosslinking buffer in a temperature controlled room at 23 °C.

All data was collected in a MiniTweezers instrument^39^. Force-ramp data was collected at a sampling rate of 200 Hz and a pulling speed of 75 nm/s. The tether stiffness, expressed in units of pN/s, is related to the loading rate (pN/s) and pulling velocity (nm/s) by Equation 1:

1 $${k}_{stiffness}=\frac{Loading\,rate\,}{Pulling\,rate\,}$$

The loading rate at which the target protein unfolds corresponds to the slope of the force-ramp data immediately before the unfolding event.

Unfolding force probability distributions obtained from force-extension curves were transformed to folded state lifetimes as a function of force and analyzed using the methodology described by Dudko *et al*.^36^. We determined the position fluctuations (mean ± standard deviation) for each dsDNA handle length as a function of force, between 4–20 pN. The position fluctuation was obtained from at least five different molecules.

## Electronic supplementary material

Supplementary Information
