# Supplementary material for: Integrated Method to Attach DNA Handles and Functionally Select Proteins to Study Folding and Protein-Ligand Interactions with Optical Tweezers
Source: Sci Rep. 2017 Sep 7;7:10843. doi: 10.1038/s41598-017-11214-z (PMC5589850; doi:10.1038/s41598-017-11214-z)
Supplement: Supplementary file 1 — Supplementary Information [file 41598_2017_11214_MOESM1_ESM.pdf]

SUPPLEMENTARY INFORMATION

for

Integrated Method to Attach DNA Handles and Functionally Select Proteins to Study Folding and Protein-Ligand Interactions with Optical Tweezers

Yuxin Hao[1], Clare Canavan[1], Susan S. Taylor[2] and Rodrigo A. Maillard[1,*]

[1] Department of Chemistry, Georgetown University, Washington, DC 20057

[2] Department of Pharmacology & Department of Chemistry and Biochemistry, University of California, San Diego, La Jolla, CA 92093

[*] Corresponding author: Rodrigo A. Maillard, Department of Chemistry, Georgetown University, 37 & O St, NW, Washington, DC 20057;

Telephone: 2027847146; Email: rodrigo.maillard@georgetown.edu

This PDF file includes

- Supplementary Figure S1
- Figure legend for Figure S1

Supplementary Figure S1

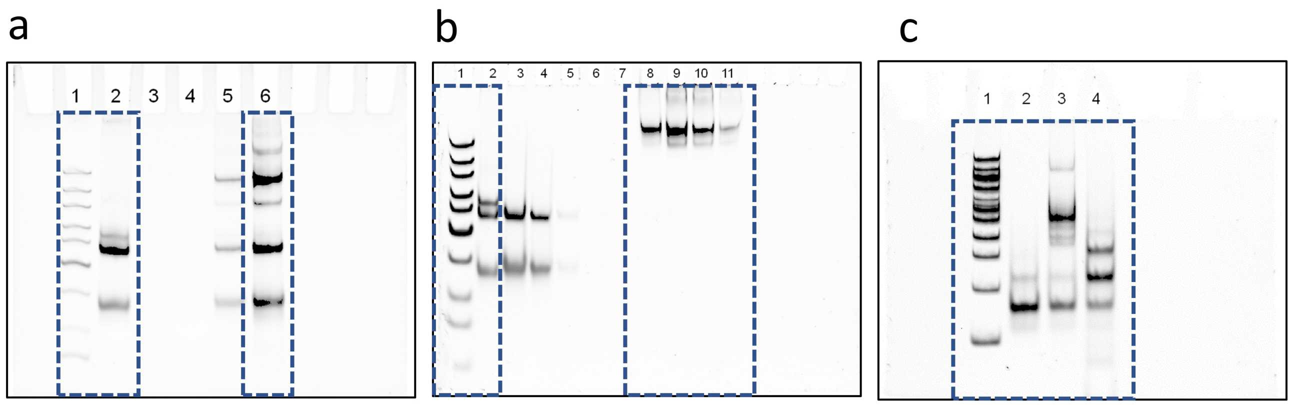

Supplementary Figure S1. Complete Images of Native Gel Electrophoresis. The lanes with dashed rectangles were selected for the Figure 2a, 2b, and 2c. Other lanes were omitted for simplicity. **(a)** 5' Thiol-modified dsOligos were attached to the CBD-A. 15% native acrylamide gel stained with ethidium bromide (EtBr). Lane 1: GeneRuler ultra low range DNA ladder (Thermo Fisher). Lane 2: dsOligo loading control. Lanes 3 and 4: empty. Lane 5: 5X dilution of 1 μL from the dsOligo attachment reaction with the protein. Lane 6: 1 μL from the dsOligo attachment reaction (due to the high efficiency of the reaction, the overexposure was difficult to avoid). **(b)** The protein-oligo chimera was functionally selected and eluted from a cAMP-coupled agarose using an increasing concentration of cAMP: 0.02 mM, 0.2 mM, 2 mM and 20 mM. 15% native acrylamide gel stained with EtBr. Lane 1: GeneRuler ultra low range DNA ladder (Thermo Fisher). Lane 2: 1 μL of flow-through after binding the protein-oligo chimera to the cAMP-coupled agarose resin (i.e., unreacted dsOligo). Lane 3-6: 1 μL of washes 1 thru 4 using 20X buffer volumes relative to the resin. Lane 7: Empty. Lane 8: Elution 1 with cAMP concentration of 0.02 mM. Lane 9: Elution 2 with cAMP concentration of 0.2 mM. Lane 10: Elution 3 with cAMP concentration of 2 mM. Lane 11: Elution 4 with cAMP concentration of 20 mM. **(c)** The selected protein-oligo chimera was ligated with long (370 bp) or short (30 bp) dsDNA handles modified with digoxigenin and biotin. 5% native acrylamide gel stained with EtBr. Lane 1: 100 bp DNA ladder (NEB). Lane 2: protein-oligo chimera only. Lane 3: protein-oligo chimera ligated with long dsDNA handles. Lane 4: protein-oligo chimera ligated with short dsDNA handles.
